# Proposal for Modeling Motivational Strategies for Autonomy Support in Physical Education

**DOI:** 10.3390/ijerph18147717

**Published:** 2021-07-20

**Authors:** Juan Antonio Moreno-Murcia, Julio Barrachina-Peris, Manuel Ballester Campillo, Estefanía Estévez, Elisa Huéscar

**Affiliations:** 1 Sport Research Center, Sport Sciences Department, Miguel Hernández University of Elche, Avda. de la Universidad, s/n, 03202 Elche, Spain; j.moreno@umh.es; 2 Sport Research Center, Miguel Hernández University of Elche, Avda. de la Universidad, s/n, 03202 Elche, Spain; juliobarrachina@gmail.com (J.B.-P.); manuel.ballester2@murciaeduca.es (M.B.C.); 3 Department of Health Psychology, Miguel Hernández University of Elche, Avda. de la Universidad, s/n, 03202 Elche, Spain; eestevez@umh.es; 4 Sport Research Center, Department of Health Psychology, Miguel Hernández University of Elche, Avda. de la Universidad, s/n, 03202 Elche, Spain

**Keywords:** motivation, interpersonal style, self-determination, physical education, adolescents

## Abstract

The motivational style that teachers adopt during their interactions with their students in class can have a significant influence on the search for optimal and balanced development. Knowing the role of motivation in generating positive change, the key is to define the strategies that constitute an adaptive motivational style of teaching. The aim of this study was to design and validate the set of motivational strategies to support autonomy that are framed within the Self-Determination Theory in the context of physical education classes. For this purpose, a five-phase process was designed and carried out in one study involving different samples of experts, teachers and students. On the one hand, 25 autonomy-supportive motivational strategies were obtained and organized according to their perceived difficulty. We also analyzed the importance attributed by teachers and the difficulty of implementing them, as well as the autonomy support perceived by students through these strategies. The results obtained made it possible to present a behavior-optimizing solution consisting of a progression of 25 autonomy support strategies. The results obtained are discussed in terms of their value in the design of educational scenarios that promote high-quality student motivation.

## 1. Introduction

One of the main factors involved in the quality of the teaching–learning process is the ways in which teachers interact with their students [1,2], which can influence the results with respect to their involvement in the tasks, as well as the quality of their motivation. To this end, the Self-Determination Theory by Deci and Ryan ((SDT) [3,4]) differentiates between two types of motivation: autonomous and controlled. Autonomous motivation is the one that is associated with better results for learning, and it takes place when the student engages in tasks for his own enjoyment of carrying them out. Meanwhile, controlling motivation occurs when students perform tasks under pressure or external threats, even to obtain rewards or avoid punishment. SDT further states that the increase in autonomous motivation in students is determined by the degree of their involvement in their own decisions, through their ability to interact with the environment and with the rest of society through their own will. In this way, the authors established that the relationship of the person with the different social contexts and its link with self-determined behavior is sustained by three main pillars [3,4]: autonomy (feeling of being the originator of decisions), competence (feeling of security and fulfilment) and the relationship with others (perceiving oneself as a member of a group and feeling integrated within it). These three states can be fostered when the social context supports these fundamental psychological needs. In this sense, the interpersonal style of autonomy support is presented as a facilitator of higher levels of intrinsic motivation in students, focusing them towards a self-determined behavior that can extend the positive results derived to different contexts [5,6,7,8]. Providing students with a learning environment that supports their autonomy structure before and during the development of tasks and involves them in their own learning by promoting relationships with others is associated with autonomous motivation and with positive results such as well-being or self-efficacy [3,4]. Thus far, despite the large number of studies that have demonstrated the suitability of this motivational style in the search for an optimized teaching–learning scenario, no previous works have focused on understanding the motivational strategies that constitute this style, in terms of the criteria associated with their validity, and the qualitative distribution of the strategies with respect to the progression that the teacher should adopt when using them to ensure their approach to an autonomy-supportive style. Moreover, with regard to this last aspect, there are no studies that help us to understand the differences in terms of the relative contribution of each strategy (importance) to the achievement of the final style of autonomy support, as well as the level of difficulty (complexity) for its implementation in the classroom. For this reason, and given the need to systematize the behaviors that teachers would need to deploy in order to develop an autonomy-supportive style with their students, this study had two objectives. On the one hand, based on previous studies [9,10], the aim was to design and validate the strategies of the physical education teacher’s autonomy support style. The second objective was to analyze the criteria of importance and the difficulty of these strategies in order to establish a final proposal for their progressive implementation in the classroom.

### 1.1. Motivational Strategies in the Teaching Process

Recent studies have corroborated the idea that when teachers employ motivational strategies that support students’ basic psychological needs, positive outcomes on motivation are optimized [11]. Thus, for example, teachers can facilitate the need for autonomy by using strategies such as offering students different options of choice, fostering relevance and using informational rather than controlling communication. For their part, to promote the need for competence, teachers should offer clarifying, positive but non-evaluative feedback, acting as a guide during the learning process, by providing structure. Finally, the teacher can facilitate the need for a relationship through cooperative work among students, promoting positive social relationships between them and acting as a positive role model for them. Further, teachers can support this need by showing affection, attuning and interest to students. This appropriateness of strategies allows teachers to guide the teaching–learning process towards better motivation and a positive predisposition for the continued use of autonomy support [12].

### 1.2. Interpersonal Style of Autonomy Support

The greater the student’s self-determined behavior, the greater their participation in the teaching–learning process, which generates a greater involvement and creates a motivational climate that engages them in learning [13]. In accordance with these arguments, teaching actions should be aimed at improving the student’s involvement, so that their behavior is oriented towards satisfying their needs for autonomy and competence [14]. In this sense, the autonomy-supportive interpersonal teaching style is characterized by taking into account the student as an essential element of the educational process for making decisions, reaching consensus and ceding responsibility, providing explanations about instruction and adapting tasks, making performance levels more flexible and generating a positive/comfortable learning context/environment based on mutual trust. In contrast, when the teacher employs a controlling style, his or her behavior puts pressure on the learner through the use of authoritarian language, articulating hermetic teaching sequences that prioritize content and outcomes over processes in instruction. This style has a negative impact on the student’s intrinsic motivation, frustrating their basic psychological needs and resulting in maladaptive outcomes [15,16,17,18]. Thus, different studies [19,20] have shown that learners feel more fulfilled and motivated when teachers employ an autonomy-supportive interpersonal style, as opposed to a controlling one [21,22,23].

In this sense, the teacher plays a momentous role within the teaching–learning process, since they could favor the increase in intrinsic motivation of the students through adequate training and the use of a self-determined motivational style that is focused on supporting autonomy, thus allowing them to improve their relationship with them and increase their perceived competence and well-being, among other outcomes [24].

### 1.3. The Present Study

Based on the above arguments, the aim of this study was to design and validate motivational strategies for autonomy support at a trans-contextual level. The project was approved by the Project Evaluation Body of the principal investigator’s university (2017.06.259.E.OEP; 2017.07.305.E.OEP; 2018.333.E.OEP). The steps needed to achieve this objective were: (a) Determine the content validity by means of expert judgement using the Delphi method [25]; (b) Confirm the validity of the understanding of the strategies; (c) Examine the difficulty in the implementation of the strategies; (d) Test the effect of the implementation of the strategies on student perception; and (e) Propose an organized sequence of actions for implementing the motivational strategies to support autonomy.

**Figure A1:**
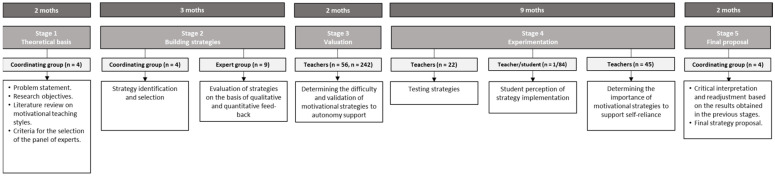
Stages and phases of the strategy measurement model.

For this purpose, the following steps in the validation process were carried out (Figure A1).

1. Theoretical foundation stage. The theoretical foundations of the model to be developed were established, as well as the characteristics of the program, based on the Self-Determination Theory [26] and on the interpersonal motivational style of autonomy support [27].

2. Strategy construction stage. The strategies to be carried out were constructed through the Delphi method, giving rise to two phases: (1) A coordination group selected a set of strategies through the bibliographic review and the existing literature, considering the basic psychological needs (competence, autonomy and relationship with others); (2) A group of experts organized the strategies proposed in the previous phase according to their difficulty and importance when putting them into practice.

3. Assessment stage. This was named step 3. A group of teachers was consulted on their assessment of the importance of the implementation of autonomy support strategies and to another group to confirm validation.

4. Experimentation stage. This was divided into three phases: (1) In order to examine the difficulty in the implementation of the strategies, a study of the implementation of the strategies was carried out; (2) In another study, the perception of the students was analyzed once the strategies had been implemented and put into practice; (3) A group of teachers was consulted and asked about their assessment of the importance of the implementation of the autonomy support strategies.

5. Final proposal stage. The coordination group, after critical analysis of the results of the previous phases, established a final proposal of strategies.

## 2. Materials and Methods

### 2.1. Ethics Statement

This study has been approved by the Research Ethics Committee of Universidad Miguel Hernández de Elche (Elche, Spain) (DPS.JMM.01.17) and meets all ethical and legal standards that are applicable to the research of this survey modality.

### 2.2. Participants

#### 2.2.1. Phases 1, 2, 3 and 5

The coordination group was composed of four people (3 men and 1 woman) who were experts in autonomy support, aged between 42 and 52 (*M* = 43.6; *SD* = 3.4), everyone with extensive experience in the field of teaching and research (*M* = 19.11; *SD =* 4.3). All of them had a situation and personal resources that allowed them to contribute positively to the achievement of the objective, being able to provide relevant input [25].

In order to obtain content validity by means of expert judgement (expert group 1), a purposive sample of 9 university teachers (5 women and 4 men), experts in autonomy support, aged between 41 and 64 (*M* = 45.7; *SD =* 6.2) and with extensive experience in the field of teaching and research (*M* = 16.81; *SD =* 7.4), was selected. All of them worked as researchers at different Spanish universities.

In order to test the difficulty of implementing the strategies, a group of teachers (expert group 2) was used. This group consisted of 56 teachers (25 women and 31 men) aged between 38 and 57 (*M* = 42.8; *SD* = 9.1) and with extensive teaching experience (*M* = 12.56; *SD =* 8.7). All of them worked as teachers in Compulsory Secondary Education and/or Baccalaureate in Spanish schools.

In order to perform a confirmatory factor analysis of the measures, a group of 242 teachers (115 women and 127 men) aged between 25 and 49 (*M* = 38.5; *SD* = 6.2) and with extensive teaching experience (*M* = 10.45; *SD =* 6.3) was used. All of them worked as teachers in Compulsory Secondary Education and/or Baccalaureate in Spanish schools.

#### 2.2.2. Phase 4

In order to implement the progression of the 25 autonomy support strategies, a sample of 22 teachers (10 women and 12 men), aged between 32 and 56 years (*M* = 46.70; *SD* = 12.20) and with extensive teaching experience (*M* = 8.56; *SD* = 12.78), was selected.

To test the effect on student perception after the application of motivational strategies, a quasi-experimental study was carried out with a sample of 84 Spanish students in the first year of Compulsory Secondary Education, aged 12–14 years (*M* = 12.5; *SD* = 1.90), of whom 45 were boys and 39 girls.

In order to test the importance of the 25 autonomy support strategies, a sample of 45 professionals (15 women and 30 men), aged between 41 and 51 years old (*M* = 46.22; *SD* = 5.89), and with extensive teaching experience (in years, *M* = 9.67; *SD* = 6.31), was selected.

### 2.3. Procedure

#### 2.3.1. Phases 1, 2 and 3

The Delphi method, based on a panel of experts, was used to collect the information for this study, and included the following sections in the structure of the schedule followed to determine content validity through expert judgement (Figure A1):

(1). Theoretical foundation stage. The selection, construction and contact with the groups of experts (experts 1 and experts 2) was carried out, so that their contribution would favor the study. The coordination group reviewed the literature on Self-Determination Theory (SDT) [9], focusing the search on the interpersonal style of autonomy support. The Preferred Reporting Items for Systematic Review and Meta-Analysis for Scoping Reviews (PRISMA) diagram [28,29] was used. The interval of analysis was limited to the last ten years, and the studies that considered the styles as two independent factors of the motivational teaching style were analyzed [18]. The following databases were consulted: Science Direct, Scopus, Psyinfo, Web of Science, Medline and TPSR Alliance. In the first stage (Identification stage), 89 articles were found in the databases and 26 were excluded by duplicate (Screening stage). After reading all titles and abstracts, 58 articles were included for reading in full, in order to answer the research question (Eligibility stage). Studies that did not meet the inclusion criteria (n = 27) were excluded due to different contexts (n = 12), different populations (n = 11) and others (n = 4). Therefore, 31 articles made up the final sample of this scoping review (Included stage).

(2). Strategy construction stage. The strategies were designed and created by the coordination group (strategies based on the bibliography and literature from stage 1) and sent to the experts for review. In order to check their external validity, the content validity technique was used by means of expert judgement. Nine university teachers, experts on the style of autonomy support, were contacted, and the aim of the study was explained to them. All of them had an extensive literary production (publications of scientific articles, direction of theses, participation in congresses, etc.) and broad experience in the technique of systematic observation. They were sent an e-mail with the context of the study and a sample of the scale so that they could rate, according to a Likert scale ranging from 1 (does not meet the criterion) to 4 (high level), the degree of sufficiency (the items belonging to the same dimension are sufficient to obtain the measurement of this dimension), clarity (the item is easily understood, i.e., its syntax and semantics are adequate), coherence (the item has a logical relationship with the dimension or indicator it is measuring) and relevance (the item is essential or important, i.e., it should be included) of the items proposed in each of the four dimensions proposed.

The experts were given sufficient time to carry out the review (approximately one month) and to provide any comments they considered appropriate. During the following month, all the guiding comments and suggested adjustments were collected, and modifications were made. After the analysis of the revisions provided by the experts, the strategies were restructured, adding and modifying some of them. After this phase, the strategies consisted of a total of 25 strategies, grouped into a construct with four dimensions: autonomy, with five strategies (e.g., “ask the student about his/her preferences in relation to a task”); structure before the task, with five strategies (e.g., “at the beginning of the lesson explain and rationalize the objectives”); structure during the task, with eight strategies (e.g., “adapt the instructions according to the students’ progress”) and relatedness, with seven strategies (e.g., “use empathetic language”). Once all the suggested adjustments had been made, the set of strategies was sent back to the experts for a second review. After this, the strategies were grouped into a single construct as shown in Table A1.

(3). Assessment stage. In order to be able to design the implementation of the strategies’ progression, a group of teachers was asked to rate the difficulty in implementing the strategies in practice. The difficulty dimension is related to the amount of resources that the teacher requires to mobilize to implement the strategies. These resources are associated with the personality and the manner of addressing the student during instruction, and include manifestations of teaching behaviors of all kinds, both quantitative and objective—for example, the use of certain materials, the frequency with which a student is addressed in a certain manner, ways of organizing the class, the range of freedom proposed for solving the tasks, etc. However, it also, and simultaneously, encompasses qualitative and subjective aspects of teaching behavior, such as the degree of empathy with students, a favorable predisposition towards the resolution of conflicts and doubts, closeness and enthusiasm shown in the development of the class, climate of trust generated, etc. In short, the difficulty factor sought to measure whether the use of certain resources by the teacher had a positive balance in the cost–benefit ratio for the development of self-determined student behavior.

To encourage reflection on the subject, a table was presented which contained each of the four main dimensions or categories of the teaching style, accompanied by a brief description. Then, all the strategies were shown so that the degree of difficulty could be rated according to a Likert-type scale ranging from 1 (*not at all important, not at all difficult*) to 5 (*very important, very difficult*).

Since we started, a priori, from an adequate theory that allowed structuring the dimensions, it was necessary to confirm that this structure could also be obtained empirically. Therefore, we proceeded to explain the covariances or correlations between a set of observed or measured variables through a reduced set of latent variables or factors, by means of a confirmatory factor analysis. For the collection of information, the physical education teachers involved were contacted to inform them of the objective of the research and to request their collaboration. They were sent the strategies to be assessed through a Likert-type scale ranging from 1 (*Surely not*) to 7 (*Surely yes*). These were sent through Google Docs questionnaires in most cases or in paper format for the participants with more direct contact. It took approximately 10 min to complete the questionnaires and the participants were assured of data privacy.

#### 2.3.2. Phase 4

In this phase, the difficulty of the 25 strategies obtained in stage 3 from the previous study was tested in different contexts (physical education, sports and health). Teachers and coaches were asked to implement the strategies progressively during their classes over a period of six weeks, so that during the first week, the first four strategies were implemented; during the second week, the first four strategies plus the next four were implemented, and so on cumulatively, until the last (sixth) week, where the 25 strategies were implemented in a comprehensive manner, incorporating the five strategies that had not been implemented previously in this last week. At the end of each week, teachers and coaches were asked to provide a weekly quantitative and qualitative evaluation of the strategies.

In addition, the week prior to the implementation of the weekly strategies (four strategies each week for the first five weeks, and five strategies on the sixth week), each expert had to develop the strategies within his or her field, making practical examples (Table A3), which were supervised by the coordination group, thereby allowing for continuous feedback between the coordination group and the experts.

Prior to this, teachers were trained in the autonomy support style. The training process replicated the models proposed in the literature for autonomy support [14,18,19,30,31,32,33,34]. Conceptual foundations and strategies for its development were explored. Several case seminars were held on the Self-Determination Theory (SDT) [4,33,35], the Hierarchical Model of Intrinsic and Extrinsic Motivation [36,37,38] and the Achievement Goal Theory [4]. The motivational orientations of the autonomy-supportive interpersonal teaching style and the controlling style were studied [18,27,39,40,41,42,43,44,45]. The proposed strategies were analyzed using several classes. The analysis was carried out separately twice a week. Thus, the intra-measurement reliability could be verified. Several training sessions were necessary to achieve an inter- and intra-observer reliability of 93.4%. This phase lasted approximately one month. Following the contributions from different authors [46,47,48], it was determined that a minimum of 80% of the total number of interactions recorded should occur under the autonomy-supportive style.

First, the school management team was contacted and the aim of the research was explained to them. Furthermore, permission was requested from the school council for the participation of the corresponding classes. In addition, due to the age of participants (underage), their parents/guardians were asked to sign a consent form for their children’s participation in the study. All participating students were treated according to institutional ethical guidelines regarding consent, confidentiality and anonymity of responses.

A quasi-experimental design was used for the selection of the sample, since the participants could not be selected randomly as they had been previously divided into groups. The entire sample was divided into three groups with a teacher who followed a model of intervention in support of autonomy. Both at the beginning and at the end, the students answered the questionnaires described above, in a period of time lasting between 10 and 15 min, depending on the speed of the class.

Prior to the implementation of the project, the teacher received training through an Autonomy Support Intervention Programme (PIAA) [24]. The intervention of the teacher who followed the PIAA model consisted of gaining interest in teaching and in the students’ learning, being positive, being patient and listening to the students, giving more importance to the process than to the final results in the tasks, respecting the differences between the different students, their learning rhythms, behaviors and interests, demonstrating empathy and adequately managing emotions during conflicts.

The intervention took place during the months of March, April, May and June, with two 50 min classes per week (24 classes in total). A proposal was made to contemplate autonomy support strategies in the progression of known difficulty. The implementation was structured progressively (four new strategies were incorporated every two weeks) with the aim that, by the twelfth week, they would all be set in place. According to Perlman [46], it is necessary to provide a minimum of 80% of the autonomy-supporting information, and for this purpose, class filming was carried out every two weeks (Table A5). A measurement instrument [48] was used to check the types of verbal interactions of the teacher (six classes were recorded, one every two weeks), and the percentages devoted to each style in their classes (autonomy-supportive or controlling style) were coded. It can be observed (Table A5) that from the fourth class onwards, the teacher managed to maintain the percentage of 80% of autonomy-supportive behavior until the end of the intervention.

Similar to what was described in phase 3, a group of teachers was asked to rate the importance of applying these strategies in practice. They were presented with a table containing each of the four broad dimensions or categories of teaching style accompanied by a brief description. Then, in the back row, all the autonomy-supportive strategies were displayed so that the degree of importance could be rated using a Likert-type scale from 1 (not at all important, not difficult) to 5 (very important, very difficult).

#### 2.3.3. Phase 5

The coordination group, after critical analysis of the results of the previous phases, and once the desired stability was obtained, established a final proposal of strategies.

### 2.4. Measures

#### Phase 2 and 3

The achievement of the implementation of the strategies was evaluated using a Likert-type scale with values from 0 (not achieved at all) to 10 (fully achieved) for each strategy. In addition, an open-ended question was incorporated to qualitatively evaluate the achievement of the implementation of the strategy (Would you like to comment on the implementation of the strategy? (obstacles, usefulness, proposal for readjustment, etc.)).

Motivational strategies for autonomy support. The 25 strategies were grouped into five items for autonomy support (e.g., “Ask the student about his or her preferences regarding a task”), another five items for structure support before the task, eight items for structure support during the task (e.g., “Adapt instructions according to students’ progress”) and seven items for relationship support (e.g., “Employ empathetic language”). It was preceded by the statement “In your physical education classes...”. It was measured on a Likert-type scale ranging from 1 (Surely not) to 5 (Surely yes).

Autonomy support. The Autonomy Support Scale (EAA) by Moreno-Murcia, Huéscar, Andrés-Fabra and Sánchez-Latorre [49] was used. This scale is composed of 12 items that measure, through a single factor, the students’ perceived need for autonomy support from their teacher in physical education classes. The items (e.g., “He explains to us why it is important to do the tasks”) were developed after the previous statement “In my physical education classes, my teacher...”. It was measured on a Likert-type scale ranging from 1 (Surely not) to 5 (Surely yes). Internal consistencies at pretest and posttest were 0.84 and 0.83, respectively.

Teacher social support. The Interpersonal Behavior Scale (IBS) by Pelletier et al. (2008) [50], validated in the Spanish context by Moreno-Murcia and Corbí [51], consisting of 12 items, was used to assess the social support of teachers by measuring the following three constructs: support for autonomy (e.g., “provides me with many opportunities to make personal decisions about what I do”), support for competence (e. g. “conveys to me that I am capable of learning”) and support for the relationship between teachers and their colleagues (“provides me with many opportunities to make personal decisions about what I do”), support for competence (e.g., “conveys to me that I am capable of learning”) and support for relationships with others (e.g., “enjoys spending time with me”). The previous statement was “My physical education teacher...”. A Likert scale ranging from 1 (Never) to 5 (Always) was used. Internal consistencies at pretest and posttest were 0.73 and 0.72 for autonomy, 0.70 and 0.81 for competence and 0.74 and 0.75 for relationship with others, respectively.

### 2.5. Data Analysis

The qualitative data were analyzed using content analysis. Regarding the quantitative data, the preparatory data analysis, the calculation of descriptive statistics and the estimation of internal consistency were performed. Descriptive analyses of strategy ratings and qualitative analyses of teachers’ contributions were carried out. Moreover, in phase 3, to obtain evidence of the reliability and validity of the designed scales, Crombach’s alpha was calculated and a confirmatory factor analysis was carried out. The estimation method used was Mean and Variance Weighted Least Squares, since the observable variables, i.e., the items, were categorical in nature. In phase 4, Cronbach’s alpha coefficient was used to check the internal consistency of each factor. The effect of the intervention was assessed through a 2 × 2 (group × Time) repeated-measures analysis (ANOVA). To answer the research questions, a repeated-measures ANOVA was conducted with all dependent variables (autonomy support and teacher social support). Data analysis was performed with the SPSS 23.0 statistical software.

## 3. Results

### 3.1. Results Stage 1. Theoretical Foundation

At this stage, the foundations were laid to shape the coordination group, which had the responsibility of defining the research problem, and we contacted the group of experts to obtain their commitment to collaborate. Among other functions, this group was responsible for interpreting the partial and final results of the research study and for monitoring the research, and they were able to make adjustments and corrections. This group consisted of four experts who met the above criteria.

The study was based on a bibliographic review, limiting the time interval of analysis to the last ten years. The lines of research were directed towards various interests. On the one hand, we analyzed the studies that dealt with teacher training prior to the implementation of the autonomy-supportive style and the perception of teachers and students regarding it. Then, we reviewed the studies that proposed the analysis and measurement of the teachers’ interpersonal style of autonomy support and/or control in different contexts. For this, special interest was paid to those that focused on the study of both styles, understood as two independent factors of the motivational teaching style [18]. In addition, the process of theoretical grounding was complemented with the review of articles presented by expert authors in the field, during the same time period.

Using as a reference the scenarios described by Reeve [27] and Reeve et al. [52] for teaching based on a motivational style, oriented towards the development of control or towards autonomy support, strategies were described based on the proposal from Barrachina, Huéscar and Moreno-Murcia [9], for the measurement of the teacher’s interactions during the approach and development of the tasks. After reviewing the conceptual delimitation proposed in the literature [14,18,29,30,31,32,46,47,48,52,53,54,55,56,57], the construct designed was narrowed down to the following dimensions of analysis: autonomy support, pre-practice structure support, practice structure support and relationship support.

Autonomy support. This is related to the teacher’s ability to generate learning environments that foster interest and take into account the preferences and personal goals of students, with the aim of promoting the mobilization of internal motivational resources, triggering the execution of tasks on their own initiative (e.g., “frequency with which he/she offers choice to the student”).

Support for structure. This is related to the creation of orderly and organized environments, as opposed to disorder, misinformation and chaos. This dimension captures all the types of support provided to the learner to help them progress in their own learning. Because of the inherent characteristics of support in this dimension, it was subdivided into factors.

Pre-task structure support. This refers to all the indications and explanations provided by the teacher about what is going to be done in a unit or session and which are offered prior to the practical development of the activities, as a prior organizer (e.g., “how often the teacher explains what is going to be done in class”). It is intended to provide guidance on what and why of an instructional process. In short, when the teacher provides pre-practice structure support, he or she aims to provide an overall understanding and to contextualize the teaching–learning processes, making them more meaningful.

Support for the structure during the task. This refers to all the instructions, guidelines, aids, feedback, praise, didactic variants or modifications, etc., that the teacher proposes in the course of the instructional process. In other words, the structure dimension corresponds to the interactions associated with the execution of a task itself, and which serve to modulate the students’ learning. They provide specific feedback on the progress made, and on how to deal with errors in a comprehensive manner. In short, it aims to develop strong practical knowledge [15], in line with the deep learner approach (e.g., “frequency with which it provides informative feedback on the outcome of an action”).

Relationship support. This refers to the generation of learning environments where the teacher is enthusiastic, has positive expectations of students and promotes trust and reciprocal affection. It refers to all the interactions in which the teacher shows empathy towards students, listens to them and tries to help solve their problems, taking into account their different points of view. In short, it seeks to establish a learning environment in which students feel respected, cared for and valued, which leads them to develop positive emotional bonds, both among their peers and with the teacher (e.g., “frequency with which the teacher listens to students with an active and positive attitude”).

### 3.2. Results Stage 2. Building Strategies

The coordination group provided a series of strategies to expert group 1 for review and optimization, which they assessed quantitatively using a Likert-type scale (1–5), while they qualitatively indicated any relevant aspects of each of the strategies proposed. In this way, the different strategies could be readjusted so that the final result was composed of 25 strategies for implementing autonomy support, covering its four domains (Table A1): autonomy support, pre-task structure, on-task structure and relationship support.

### 3.3. Results Stage 3. Assessment

Once the results from stage 2 were obtained, an assessment of the difficulty of the autonomy support strategies of the previous phase was provided to expert group 2, ultimately obtaining, as shown in Table A2, 25 autonomy support strategies, listed as a progression from the least to most difficult.

In the confirmatory factor analysis and internal consistency of autonomy support, the χ^2^ test value and fit indices for the model consisting of four factors were as follows: χ^2^(59, 266) = 589.35 (*p* < 0.001), CFI = 0.90, RMSEA = 0.05 [0.05, 0.06]. Factor weights ranged from 0.33 to 0.81. Furthermore, internal consistency for autonomy support was 0.76, for pre-training structure was 0.89, for structure during training was 0.84 and for relationship support was 0.91.

### 3.4. Results Stage 4. Experimentation

In order to check the difficulty of the 25 autonomy support strategies, the level of achievement of each of the strategies by each of the experts was measured, both quantitatively (Table A4) and qualitatively. At the qualitative level, and after analyzing the open questions asked by the experts, it was found that there was a difficulty in implementing the first four strategies (first week) related to maintaining the students’ attention. With strategies 5, 5, 7 and 8 (second week), all the teachers indicated good internalization and ease of implementation. With regard to strategies 9, 10, 11 and 12 (third week), good feasibility was described when putting them into practice, highlighting, above all, positive reinforcement and being enthusiastic. Finally, the last five autonomy support strategies showed high feasibility for implementation, highlighting the gratitude of the process carried out, by both teachers and students.

To test the effect of the implementation of the autonomy support strategies on the student, the autonomy support perceived by the students was measured, in addition to the social support given by the teacher by means of self-reports. After performing the repeated-measures ANOVA, and after the intervention with the motivational strategies, the student’s perception of autonomy support (M pre = 3.23 and M post = 3.76, *p* < 0.01), social support for autonomy (M pre = 3.01 and M post = 3.66, *p* < 0.01), social support for competence (M pre = 3.02 and M post = 3.26, *p* < 0.01) and social support for relatedness (M pre = 2.72 and M post = 3.38 *p* < 0.01) improved.

### 3.5. Results Stage 5. Final Proposal

The main objective of this phase was to be able to readjust the motivational strategies for autonomy support, based on the results from all the phases of the study. After analyzing the criteria of a group of experts on autonomy support through the difficulty involved in the implementation of these strategies (phase 3), checking the achievement of the objectives of the strategies in different contexts (phase 4) and verifying their effectiveness in the pupils (phase 4), the differences between the initial proposal of difficulty progression, and the results of their implementation, became evident. Concerned with this, we asked for an evaluation of the importance of the strategies (phase 4), and we found, almost across the board for all 25 strategies, that there was an almost inverse relationship between the difficulty of the tasks and the importance of each one of them.

In relation to the analysis of the importance of the 25 autonomy support strategies (Table A6), it is worth noting that the autonomy support strategies were found in higher positions, with the exception of one of them, which was located at the end of the ratings (13. Ask the learner about his/her preferences in relation to a task). On the other hand, both the on-task structure strategies and the relationship support strategies were found in intermediate positions in relation to the mean importance. The pre-task structure strategies were the ones that, as a general rule, obtained lower scores (except for strategy 21. To offer guidelines and orientations to regulate personal progress and to make the criteria for improvement known in advance, which was among the three highest scoring strategies).

In order to make this adjustment, and with the aim of increasing the motivation of the professionals to put them into practice, a new categorization of the implementation of the strategies was performed, organizing them into three phases, where the simplest strategies are presented in the first phase; in the intermediate phase, the most difficult strategies are developed, and then in the third phase, the tasks with less difficulty than in the previous phase are put into practice. In addition, during the first phase, it was also a criterion that strategies with high importance should be present. For this purpose, a cut-off point of difficulty was established both in the results from study 1 and in the results from phase four of study 2 with regard to importance. This cut-off point was determined by the mean (M) of the scores obtained in both results (Study 1, *M* = 1.72; Study 2, *M* = 8.37). After considering all of these criteria, the first phase consisted of six strategies, phase two of 11 strategies and phase three of eight strategies (Table A7).

## 4. Discussion

The aim of the present work was to design and validate the set of motivational strategies of autonomy support framed within the Self-Determination Theory in the context of physical education classes. Thus, a restructuring and reorganization of the autonomy-supportive motivational strategies, based on the experiences of different professionals, would be better adjusted to the real practice needs. This study provides teaching experts with a proposal for a more adjusted and outlined progression by which to achieve an interpersonal style of autonomy support, which seeks to satisfy basic psychological needs [17,18,24,58].

For this purpose, the modeling of the strategies was structured into two major studies, which were subdivided into five stages. During stage 1, the coordination group defined the first problem statement, establishing the research objectives and selecting the expert groups. Subsequently, and within the first study, stage 2 was divided into two distinct phases: on the one hand, the coordination group established a number of autonomy support strategies, while on the other hand, a group of experts validated these strategies, resulting in a total of 25 autonomy support strategies. In the third stage, a group of experts determined the difficulty in implementing these strategies. Confirmation of the scale at the factorial level was also carried out with satisfactory indices. In stage 4, a group of teachers put the 25 autonomy support strategies into practice to obtain the degree of difficulty in the implementation of these strategies. In another phase, the perception of the implementation of an autonomy-supportive interpersonal style was tested by a group of students, while in the last phase, the level of importance of each of the 25 motivational strategies was tested by a group of teachers. Then, taking into account the results of the two studies, in order to be able to show an optimal final proposal, the 25 autonomy-supportive strategies were readjusted and restructured.

Due to the importance of the teacher considering social support strategies to promote the satisfaction of psychological needs, regardless of the phase in which they occurred [1,18,29,30,32,36,46,47,48,52,53,54,55,56,57], another criterion set in this new reorganization was that strategies from the four blocks (autonomy support, pre-task structure, on-task structure and relationship support) should always be present in each phase.

Among the limitations found, and with the aim of improving the motivational strategies for autonomy support, it is necessary to continue the study to test their effectiveness with larger samples. Being able to adjust the strategies to each of the domains, it would be advisable to experiment with the application of the last proposal in different contexts. In addition, it would be useful to test whether implementing the strategies over a longer period of time would lead to better results.

This proposal suggests that this progression be put into practice over a longer period of time, allowing the teachers to internalize the strategies and to become more adaptable to the process, which may increase motivation towards a greater achievement of self-determined behavior in students [49,59,60,61].

Thus, this new proposal can be used, above all, in the field of physical education, although it could be extrapolated to the fields of specific sports and healthy physical activity. However, further studies would be necessary in the latter two fields to more specifically adjust the proposal to the needs and perceptions of the experts to create a more optimal progression.

## 5. Conclusions

In conclusion, a proposed progression of 25 motivational strategies to support autonomy is presented, which have undergone a modeling process to optimize their implementation to the greatest degree.

## Appendix B

**Table A1:** Autonomy support strategies.

Support for autonomy	Ask the learner about his/her preferences in relation to a task.
	Offer the student choice (groupings, materials and spaces).
	Letting the learner take the initiative (ceding the initiative).
	Offer possibilities for experimentation (individualize teaching).
	Assigning responsibility.
Structure before the task	At the beginning of the class explain and rationalize the objectives.
	Explain the structure of the task in relation to the class.
	Explain the usefulness of the tasks.
	Use students as positive role models for demonstrations.
	Offer guidelines and orientation to regulate personal progress and to make the criteria for improvement known in advance.
Structure during the task	Adapt instructions according to the progress of the students.
	Using role models through students.
	Demonstrations need to be shared with students.
	Propose different variations for the same task.
	Offer both verbal and non-verbal positive reinforcement. Encourage students to persevere.
	Provide informative feedback during the execution of tasks.
	Adjust the difficulty of the tasks according to the level of the students.
	Propose flexible groups according to the development of the tasks.
Relationship support	Address students in a polite and individualized manner.
	Use empathetic language.
	Listen to students with an active and positive attitude.
	Approach the student for assistance.
	Be enthusiastic.
	Give students confidence.
	Behave as a positive role model for students.

## Appendix C

**Table A2:** Difficulty rating of autonomy support strategies.

Strategies		Medium Difficulty
1	At the beginning of the lesson, explain and rationalize the objectives.	1.34
2	Approach the student for assistance.	1.34
3	Address students in a polite and individualized manner.	1.37
4	Listen to students with an active and positive attitude.	1.41
5	Give students confidence.	1.41
6	Use empathetic language.	1.42
7	Behave as a positive role model for students.	1.47
8	Provide informative feedback during the execution of tasks.	1.47
9	Explain the structure of the task in relation to the class.	1.48
10	Be enthusiastic.	1.50
11	Offer both verbal and non-verbal positive reinforcement. Encourage students to persevere.	1.50
12	Explain the usefulness of the tasks.	1.52
13	Ask the learner about his/her preferences in relation to a task.	1.54
14	Propose different variations for the same task.	1.63
15	Demonstrations need to be shared with students.	1.72
16	Use students as positive role models for demonstrations.	1.77
17	Offer the student choice (groupings, materials and spaces).	1.79
18	Propose flexible groups according to the development of the tasks.	1.80
19	Adapt instructions according to students’ progress.	1.95
20	Letting the learner take the initiative (ceding the initiative).	1.97
21	Offer guidelines and orientations to regulate personal progress and to make the criteria for improvement known in advance.	1.98
22	Using role models through students.	2.01
23	Offer possibilities for experimentation (individualize teaching).	2.02
24	Adjust the difficulty of the tasks according to the level of the students.	2.04
25	Assigning responsibility.	2.11

## Appendix D

**Table A3:** Examples of autonomy support strategies.

Strategy	Context	Teacher/Trainer
1. At the beginning of the lesson, explain and rationalize the objectives.	Before starting the main part of the session, while users warm up.	“Today we are going to improve the strength of the lower body muscles, as it has been proven that the higher the level of strength, the higher the level of health”.
2. Approach the student for assistance.	In a set-piece strategy task.	If anyone has any questions, just let me know and we’ll try to solve them.
3. Address students in a polite and individualized manner.	In the first strength session, we approached Jose Angel individually while he was warming up and we were interested on his tastes in reference to training.	“Hello Jose Angel, how are you? Today we are going to work on lower body strength, which exercises do you like the most? which ones do you like the least? which muscle groups do you like the most?
4. Listen to students with an active and positive attitude.	At the end of a physically demanding task. While the players recover by hydrating	I approach them with the aim of listening as they talk to each other about how they feel after the task and what they thought of it.
5. Give students confidence.	The talk before a match.	‘‘You have to start believing that you are good players. That you are going to win today. The week of training has been very good and I know that everything we have worked on is going to work out perfectly’’.
6. Use empathetic language.	Before performing a physically demanding task.	I know you don’t like this task. I don’t like it either. I used to get really pissed off with my coach whenever he gave us this kind of task. But throughout the season you will realize how important this preparation has been’’.
7. Behave as a positive role model for students.	During a group session, made up of a group of boys between 12 and 16 years of age and with the objective of hypertrophy. The trainer explains before the session that he tries to plan the training sessions taking into account the tastes of each one, always respecting the main objective.	“Good evening guys, before starting the session I wanted to tell you that I put a lot of effort in planning sessions with the exercises that you like the most and with which you feel more comfortable, with the aim that you have fun and have a good time, apart from achieving your goals”.
8. Provide informative feedback during the execution of the tasks.	During a training match.	“Very good Borja! That’s the clearance we worked on earlier. As you made the clearance very well, you managed to stay alone in front of the goalkeeper’’.
9. Explain the structure of the assignment in relation to the class.	Before starting a training task by addressing the whole group.	Today in the physical task we are going to perform different strength poses for 30 s. After 30 s I will whistle and you will perform a 3 vs. 2 action’’.
10. Be enthusiastic.	Talk to the group after losing a match.	Nodding his head and looking satisfied. Losing like this is not losing! You gave everything you had on the pitch. This match is going to teach us a lesson! ‘‘.
11. Offer both verbal and non-verbal positive reinforcement. Encourage students to persevere.	During a torso strength training session, the trainer proposes Trini to perform Assisted Lunges to improve the strength of her back muscles. During the session, Trini is able to do 10 repetitions without rest.	“Very good Trini, you are making great progress. You have achieved all this thanks to your effort, before you needed a rest to be able to do 10 repetitions and now you can do them without pause. This indicates that your back muscles are much stronger, you will notice that your back doesn’t hurt as often”.
12. Explain the usefulness of the tasks.	Tactical task of ball out.	The aim of this task is for you to automate the different movements you can make in a game to receive in advantageous situations so that we can get the ball out from the back with short passes.
13. Ask the learner about his/her preferences in relation to a task.	At the start of a concurrent training session, the trainer suggests the following to Ivan:	“Hi Iván, today we are going to have two quite accentuated work blocks, one where strength is the main focus and the other where endurance is the main focus, which one would you like to start with?
14. Propose different variations for the same task.	During a possession task with different goals (one large goal with goalkeeper and 3 small goals without goalkeeper) in which two teams face each other.	Once you have passed the ball 5 times between you, you can score in either goal’’.
15. Demonstrations need to be shared with students.	During a training match	Look at the way I position my body, so I can direct the ball to the other side’’.
16. Rely on students as positive role models for demonstrations.	During a group session (Ángel, Luis and Alejandro), the trainer is going to propose a training block of 6 series of 1 ’ in a coordination ladder. The trainer explains	“Ok guys, now we’re going to do 6 sets of 1’ on a coordination ladder with a 20” rest between sets. Each of you is going to be in charge of coming up with different movements that we’ve done before to do on the ladder for 2 sets. We will try not to repeat an exercise that has already been done by a colleague. Are you ready?
17. Offer the student choice (groupings, materials and spaces).	Before starting the main part of the training and with the whole group together.	“I have two tasks prepared for Saturday’s game. One is to retreat and press and the other is to counter-attack, which one do you prefer to do?
18. Propose flexible groups according to the development of the tasks.	Propose flexible groupings according to the development of the task.	Today we are going to play a match in which each team will play a different system. One will play 4-3-3 and the other will play 5-3-2. As you all know, playing with the 5-3-2 system is more difficult. Those of you who are clearer about that system will form that team and those of you who are less clear about it will play in the 4-3-3 team”.
19. Adapt instructions according to students’ progress.	In a group session, the session is laid out with repetitions, rest and loads for all exercises, set individually. The trainer says:	“Hi guys, today we are a bigger group than usual, so I have put all the information about the session written on the wall, so keep an eye on it. Take a look at it, whoever knows how it works can start and whoever has any questions can ask me and we’ll sort them out. Here we go!”
20. Let the learner take the initiative (cede the initiative).	Before performing an aerobic test (Cooper’s test)	“We are going to do an aerobic test to see what you can do. As we have done this before and you all know your old record, I would like you to set a target to try to beat and at the end of the task we will see if you have achieved it”.
21. Offer guidelines and orientations to regulate personal progress and make the criteria for improvement known in advance.	Before carrying out a possession (two teams play against each other with the aim of keeping possession of the ball) of level 3 (level 1: possession with two supporting players with superiority; level 2: possession with two supporting players with superiority in reduced space; level 3: possession without supporting players).	“Today we are going to carry out a level 3 possession. In this possession there will be no support, i.e., we will play with equal numbers. You must be very attentive and give an outlet to the ball holder because in this possession you will not have any free teammates.’’
22. Using role models through students.	When performing a Bulgarian squat, the coach observes that 2 of the 3 users perform the squat poorly technically. On the other hand, Cristian performs it very well.	“Ok guys, now that we know how to perform the exercise with great features, let’s get down to the nitty-gritty. Cristian, come out here and perform the Bulgarian Squat. You see how Cristian keeps his body upright and doesn’t lift his heel, that’s how we all have to try to do it. Great!”
23. Offer possibilities for experimentation (individualize teaching).	2vs2 action with cross and shot (An attacker will try to dribble past his defender and put a cross into the box for his teammate to shoot past the covering defender.	We are going to perform a 2vs2 task with cross and shot. In this task we are going to experience the 1vs1 that usually arises on the wing in matches and the marking inside the box’’.
24. Adjust difficulty of the tasks according to the level of the students.	In a task to improve the physical capacity of the athletes, we will divide them into 3 groups: 1st group will perform the task with a medium intensity. 2nd group with a high intensity. 3rd group with a very high intensity.	In today’s physical task we are going to divide into three groups according to your level of physical ability, so that you all work at a level that suits you’’.
25. Assigning responsibility.	In a session with a group that has some experience, the trainer will hand over the responsibility to each person to warm up according to the session to be held.	“Good morning guys, from now on I wanted to tell you that everyone will be responsible for their own warm-up. I will show you the structure of the session and then you will warm up for the first 10 min”.

## Appendix E

**Table A4:** Achievement of the objective after implementation of the strategies.

Strategies	Target Achievement (0–10)
1	8.3
2	8.6
3	8.7
4	8.4
5	8.7
6	8.7
7	8.9
8	8.8
9	7.8
10	9.14
11	9
12	8.57
13	8.31
14	8.31
15	9.08
16	9.38
17	8.18
18	8
19	8.36
20	7.82
21	7.87
22	9.13
23	8.33
24	8.53
25	8.67

## Appendix F

**Table A5:** Percentage of autonomy support and controlling style.

Classes	Autonomy Support (%)	Controlling Style (%)
1	37.8	62.5
2	45.2	55.0
3	68.8	31.2
4	85.3	15.3
5	81.4	18.5
6	82.5	17.4

## Appendix G

**Table A6:** Rating the importance of autonomy support strategies.

Strategies		Medium Importance
24	Adjust the difficulty of the tasks according to the level of the students.	4.58
25	Assigning responsibility.	4.58
21	Offer guidelines and orientations to regulate personal progress and to make the criteria for improvement known in advance.	4.44
19	Adapt instructions according to students’ progress.	4.36
15	Demonstrations need to be shared with students.	4.33
20	Letting the learner take the initiative (ceding the initiative).	4.33
22	Using role models through students.	4.24
4	Listen to students with an active and positive attitude.	4.22
17	Offer the student choice (groupings, materials and spaces).	4.22
23	Offer possibilities for experimentation (individualize teaching).	4.22
2	Approach the student for assistance.	4.20
6	Use empathetic language.	4.20
1	At the beginning of the lesson, explain and rationalize the objectives.	4.18
8	Provide informative feedback during the execution of tasks.	4.11
14	Propose different variations for the same task.	4.07
5	Give students confidence.	4.00
16	Use students as positive role models for demonstrations.	4.00
10	Be enthusiastic.	3.98
18	Propose flexible groups according to the development of the tasks.	3.91
7	Behave as a positive role model for students.	3.89
11	Offer both verbal and non-verbal positive reinforcement. Encourage students to persevere.	3.87
3	Address students in a polite and individualized manner.	3.82
9	Explain the structure of the task in relation to the class.	3.80
13	Ask the learner about his/her preferences in relation to a task.	3.67
12	Explain the usefulness of the tasks.	3.56

## Appendix H

**Table A7:** New organizational proposal for autonomy support strategies.

NO.	Strategy	Group	Phase
4	Listen to students with an active and positive attitude.	AR	First
2	Approach the student for assistance.	AR	
6	Use empathetic language.	AR	
1	At the beginning of the lesson, explain and rationalize the objectives.	EA	
8	Provide informative feedback during the execution of tasks.	ED	
13	Ask the learner about his/her preferences in relation to a task.	AA	
24	Adjust the difficulty of the tasks according to the level of the students.	ED	Second
25	Assigning responsibility.	AA	
21	Offer guidelines and orientations to regulate personal progress and to make the criteria for improvement known in advance.	EA	
19	Adapt instructions according to students’ progress.	ED	
15	Demonstrations need to be shared with students.	ED	
20	Letting the learner take the initiative (ceding the initiative).	AA	
22	Using role models through students.	ED	
23	Offer possibilities for experimentation (individualize teaching).	AA	
5	Give students confidence.	AR	
18	Propose flexible groups according to the development of the tasks.	ED	
16	Use students as positive role models for demonstrations.	EA	
17	Offer the student choice (groupings, materials and spaces).	AA	Third
14	Propose different variations for the same task.	ED	
10	Be enthusiastic.	AR	
7	Behave as a positive role model for students.	AR	
11	Offer both verbal and non-verbal positive reinforcement. Encourage students to persevere.	ED	
3	Address students in a polite and individualized manner way.	AR	
9	Explain the structure of the task in relation to the class.	EA	
12	Explain the usefulness of the tasks.	EA	

Note: AA = autonomy support; EA = pre-task structure; ED = on-task structure; AR = relationship support.
